# Beta-hydroxybutyrate ingestion with caffeine improves time to exhaustion performance following loaded exercise in the heat

**DOI:** 10.1080/15502783.2025.2550202

**Published:** 2025-08-28

**Authors:** Riccardo F. Romersi, Blaine S. Lints, Jenica N. Earl, Maria G. Lombardi, Katelynn T. Persaud, Sten O. Stray-Gundersen, Noah K. Nakagawa, Chimaobim E. Martin-Diala, Gianna F. Mastrofini, Adam T. Harrison, James E. Stampley, Shawn M. Arent

**Affiliations:** University of South Carolina, Department of Exercise Science, Arnold School of Public Health, Columbia, SC, USA

**Keywords:** Supplement, fatigue, thermoregulation, performance

## Abstract

**Background:**

Special Operations Forces operate in hot conditions under heavy loads, which impairs thermoregulation and performance and risks mission success. Exogenous ketone monoesters, such as beta-hydroxybutyrate (BHB), may enhance cerebral blood flow and metabolism and mitigate fatigue. Therefore, this study compared the effects of BHB or carbohydrate (CHO), both combined with caffeine, on a time to exhaustion (TTE) trial following prolonged loaded exercise in the heat.

**Methods:**

Using a randomized, double-blind, counterbalanced crossover design, 17male endurance-trained participants (age = 23.8 ± 5.2 y; VO_2max_ = 58.6 ± 3.2 ml/kg/min) completed two experimental trials. Exercise was performed in an environmental heat chamber (34°C, 45%RH) and included two 45-min treadmill bouts at 50% velocity at VO_2max_; (speed = 4.4 ± 0.4 mph; 5% grade) while wearing a weighted vest loaded with 20% of body mass (BM). Participants consumed 4 mg/kg BM of caffeine at the start of each experimental visit. Before each 45-min bout of exercise, participants ingested either 25 g of BHB or 25 g of CHO (Cluster Dextrin^TM^). Following the second 45-min bout of exercise, participants completed an unloaded TTE trial at 90% velocity at VO₂_max_. Heart rate and core body temperature were measured continuously using a chest strap (Polar H10, Polar Electro, Kempele, Finland) and a rectal thermistor, respectively. Experimental visits were separated by 7–14 days. Data were analyzed using paired t-tests (α = 0.05). Effect sizes were calculated using Cohen’s d.

**Results:**

TTE duration was longer in the BHB condition (9.0 ± 4.4 min) compared to CHO (7.1 ± 2.2 min; *p* = 0.03, d = 0.6). No differences were observed in average heart rates or core body temperatures between conditions (BHB: 38.72 ± 0.48°C, 186.3 ± 7.4 bpm; CHO: 38.8 ± 0.4°C, 187.7 ± 8.5 bpm; *p* > 0.05).

**Conclusions:**

BHB ingestion with caffeine improved TTE running performance in the heat compared to carbohydrate with caffeine following loaded exercise without differences in heart rate or core temperature. BHB may act as an alternative oxidative fuel, reducing glycogen reliance. Further, BHB yields more ATP per unit of oxygen than carbohydrate, which could improve energetic efficiency. These findings support the potential use of BHB as a countermeasure to fatigue during prolonged exertion in austere hot environments commonly faced by SOF personnel. Given the established ergogenic benefit of carbohydrate supplementation during exercise-heat stress, future studies should examine carbohydrate-BHB coingestion under similar conditions to explore potential synergistic effects.

